# Cardiac Arrest From Undiagnosed Catecholaminergic Polymorphic Ventricular Tachycardia: A Case Report

**DOI:** 10.7759/cureus.42190

**Published:** 2023-07-20

**Authors:** Niresh T Kuganeswaran, Michael Smith, David Chan

**Affiliations:** 1 Emergency Medicine, Carle Foundation Hospital, Urbana, USA; 2 Pediatric Cardiology, Carle Foundation Hospital, Urbana, USA

**Keywords:** case report, cardiology, emergency medicine, resuscitation, pre-hospital emergency medicine, ems, out-of-hospital cardiac arrest, pediatric cardiac arrest, cpvt, catecholaminergic polymorphic ventricular tachycardia

## Abstract

Catecholaminergic polymorphic ventricular tachycardia (CPVT) is a rare inherited heart disease in which exercise or acute emotional stress can cause potentially fatal tachyarrhythmias. We present the case of a 16-year-old female patient with a history of unexplained palpitations and syncope who suddenly collapsed in her high school cafeteria following an impassioned debate. In cardiac arrest consisting of coarse ventricular fibrillation, she was resuscitated on-scene by the school nurse via automated external defibrillation. Months later, after substantial investigation, a diagnosis of CPVT was reached. The patient made a full neurological recovery, and one year post-arrest, she was event-free on β-blocker therapy. This case demonstrates the importance of clinician awareness of CPVT, an unusual but treatable cause of cardiac arrest. Because catecholamine administration is directly contraindicated for patients with CPVT, resuscitative and post-arrest care are unique. These patients tend to be previously healthy, with normal resting electrocardiograms and no cardiac structural abnormalities, making diagnosis quite challenging.

## Introduction

Sudden cardiac arrest in children is a rare occurrence, with an annual out-of-hospital incidence of 2.28 to 18.0/100,000 person-years [1]. The survival to hospital discharge rate for those affected is 17.6-40.2%, but only 1-4% of survivors have favorable neurological outcomes [1]. Here, we report a case of pediatric cardiac arrest due to undiagnosed catecholaminergic polymorphic ventricular tachycardia (CPVT) with complete neurological recovery. This article was previously presented as a meeting abstract at the 2022 Carle Illinois Health Innovation Research Day on April 8, 2022.

## Case presentation

A healthy 16-year-old female student suddenly collapsed in her high school cafeteria following a reportedly intense discussion. Bystanders called for help, and a school nurse found the girl to be in cardiac arrest, which initially manifested with seizure-like symptoms. The nurse promptly initiated cardiopulmonary resuscitation (CPR), and after receiving three electric shocks with an automatic external defibrillator (AED), the girl regained spontaneous but shallow and irregular breathing. The AED data were later reviewed and confirmed as having coarse ventricular fibrillation. Additionally, the patient's downtime," or time from cardiac arrest onset to sustained return of spontaneous circulation, was estimated to be nine minutes. Paramedics arrived on the scene and provided manual ventilation. En route to the hospital, the patient was hemodynamically stable with a normal sinus rhythm but began to display tonic posturing and a worsening respiratory effort. Midazolam was administered as an anticonvulsant to reduce sympathetic tone. At the hospital, the emergency physician intubated the patient and ordered the administration of levetiracetam as seizure prophylaxis. A head computed tomography scan was negative for acute pathology. Patient serum was acidotic (pH = 7.235) (reference range: 7.350-7.450) with elevated lactate (3.45 mmol/L) (reference range: 0.50-2.00 mmol/L) and troponin I (0.07 ng/mL) (reference range: 0.00-0.05 ng/mL) levels, presumably secondary to recent cardiac arrest and defibrillation. A complete blood count, a urine drug screen, and a urinalysis all yielded normal results. With the exception of likely stress-induced hyperglycemia (256 mg/dL) (reference range: 60-99 mg/dL), a comprehensive metabolic panel showed no abnormalities. Abnormal laboratory findings at emergency department admission are summarized in Table 1.

**Table 1:** Abnormal patient laboratory findings at emergency department admission.

Laboratory test	Patient result	Reference range
pH	7.235	7.350–7.450
Lactate	3.45 mmol/L	0.50–2.00 mmol/L
Troponin I	0.07 ng/mL	0.00–0.05 ng/mL
Glucose	256 mg/dL	60–99 mg/dL

Patient history included anxiety and depression, which were managed with fluoxetine, resulting in a baseline electrocardiographic QTc interval of 369 milliseconds (reference range: 360-460 ms). Additionally, she had experienced two prior syncopal episodes. The first occurred while the patient was running in a track meet and was attributed to dehydration. The second event was a year later at her home, when she had become concerned for her father and sprinted onto the driveway. The cause of this more recent episode has never been determined.

**Figure 1:**
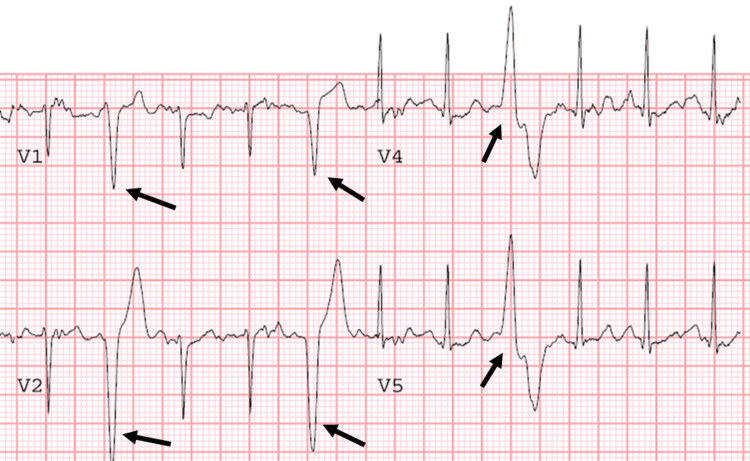
Patient electrocardiogram showing multiform ventricular ectopy (black arrows) triggered during intravenous epinephrine stress testing.

In the evening following her cardiac arrest, the girl was admitted to the pediatric intensive care unit. She was actively cooled under a targeted temperature management protocol. An electroencephalogram (EEG) was ordered to rule out ongoing seizures and diagnose a potential anoxic injury. No epileptiform activity or signs of anoxia were detected on the EEG. Suspicion consequently shifted to cardiac causes for the arrest. Furthermore, the patient's seizure-like activity from the arrest onset was accredited to arrythmia-induced cerebral hypoperfusion instead of some primary neurological event. Resting electrocardiographs (EKG) recorded regularly throughout the patient's hospitalization showed normal sinus rhythm with prominent U waves. Echocardiography was also normal. The patient was extubated to room air and then transferred to a pediatric specialty hospital for cardiac magnetic resonance (CMR) imaging and automatic cardioverter-defibrillator (ICD) implantation. The CMR revealed no structural defects within the patient’s heart. Pertinent negatives in the differential diagnosis thus included hypertrophic cardiomyopathy, arrhythmogenic right ventricular cardiomyopathy, and anomalous coronary arteries. As a secondary prevention against recurrent cardiac arrests, a Medtronic Visia AF MRI VR SureScan single-chamber ICD device was implanted. Long QT syndrome was suspected, and intravenous (IV) epinephrine stress testing was used to confirm this diagnosis. During epinephrine infusion, the patient’s EKG showed paradoxical prolongation of the QTc interval, a hallmark of long QT syndrome. This contrasted with the response typically observed in healthy individuals, where adrenergic stimulation results in a reduced QT interval and constant QTc duration. Multiform-triggered ventricular ectopy was also induced in the patient 13 minutes into the infusion (Figure 1). The patient was placed on a daily regimen of the beta-blocker nadolol (40 mg/day) as additional secondary prevention of syncope and cardiac arrest.

Three months after discharge, the patient remained asymptomatic. The diagnosis was surmised to be long QT syndrome type 1 or 2, but she had not undergone confirmatory genetic testing. A consulting cardiologist observed that the girl’s epinephrine stress test result was, in fact, a false positive. Although it appeared that an epinephrine infusion produced paradoxical QTc interval prolongation, in reality, the QTc interval was contaminated by the patient’s adolescent U wave, leading to a misinterpretation of the EKG. In addition, the cardiologist found the adrenergic induction of multiform-triggered premature ventricular contractions (PVCs) to be highly suggestive of an underlying diagnosis of CPVT rather than long QT syndrome.

Genetic screening of the patient identified a novel variant in her RyR2 gene (A3734C), classified as a probable pathogenic cause of CPVT. Variants in her KCNA5 and GAA genes were also detected, but these either did not have a clear disease association (KCNA5) or were simply benign (GAA). Outpatient treadmill stress testing revealed the development of frequent PVCs with increasing exercise, thereby verifying her new diagnosis of CPVT.

Despite the patient’s changed diagnosis to CPVT, her treatment remained the same. To reduce adrenergic stimulation, she was placed on exercise restriction, which included the avoidance of strenuous exercise and competitive sports. Her ICD remained active to ensure the conversion of any triggered ventricular tachycardia. As nadolol is considered the β-blocker of choice for CPVT therapy, she was advised to continue taking the medication at 40 mg/day. One year post-ICD implantation, the patient was event-free on 40 mg/day of nadolol.

## Discussion

CPVT is a genetic arrhythmogenic disorder characterized by emotion- or exercise-induced polymorphic ventricular tachycardia in individuals without cardiac structural defects [2]. The disease is uncommon, with an estimated prevalence of 1:10,000 [2]. Nevertheless, CPVT is still significant, as it has an untreated pediatric mortality rate of 50% [3]. Although the disease first manifests at a median age of 15 ± 10 years, CPVT is a known cause of sudden infant death [3]. Moreover, some individuals spend much of their lives asymptomatic and are only diagnosed with the disorder well into adulthood [4]. Initial clinical presentation is variable, ranging from minor symptoms of palpitations or dizziness to syncope, seizure-like activity, and cardiac arrest [4].

The pathophysiology of CPVT involves aberrant regulation of cardiac excitation-contraction coupling [3]. In healthy individuals, electrical depolarization of the myocyte membrane leads to Ca2+ influx during the second phase of the action potential. This influx subsequently triggers the release of Ca2+ stored in the sarcoplasmic reticulum (SR) through the cardiac ryanodine receptor, in a process known as calcium-induced calcium release. Free cytosolic Ca2+ then binds troponin C, and myocardial contraction can occur. Afterward, in diastole, intracellular Ca2+ concentrations decrease drastically as Ca2+ release from the SR halts, Ca2+ is transported back to the SR by a Ca2+-ATPase pump, and Ca2+ is pumped extracellularly via a Na+/Ca2+ exchanger. In CPVT-affected individuals, diastolic Ca2+ leak from the SR occurs in response to β-adrenergic stimulation, leading to delayed afterdepolarizations (DADs) [3]. Strictly speaking, elevated concentrations of intracellular Ca2+ in diastole activate the Na+/Ca2+ exchanger, which generates membrane depolarization via inward Na+ currents [5]. Suprathreshold DADs can trigger action potentials in diastole, resulting in the production of PVCs [5]. Three or more consecutive PVCs are defined as ventricular tachycardia. Sustained ventricular tachycardia can worsen coronary hypoperfusion and hence myocyte irritability, leading to ventricular fibrillation, as likely occurred with our patient.

Forms of CPVT are classified based on the mutated gene variant that led to the diastolic Ca2+ leak from the SR [3,6]. In the case we describe, the patient presents with the most common form of CPVT, known as CPVT1, in which there has been a mutation in her cardiac ryanodine receptor gene (RyR2). While CPVT1 has an autosomal dominant inheritance pattern, a recessive form of the disease also exists called CPVT2, in which the gene for the calcium-binding protein calsequestrin (CASQ2) is mutated [3,6]. Other rarer forms of CPVT exist as well, in which the TRDN, TECRL, CALM1, CALM2, or CALM3 genes are mutated [7].

In the differential diagnosis of sudden cardiac arrest without structural heart abnormalities, CPVT should always be considered along with similar conditions such as long QT syndrome, Brugada syndrome, and early repolarization syndrome [8]. Clinicians should hold a high index of suspicion for CPVT if the following features are exhibited: (i) cardiac arrest occurred during strong emotion or exercise; (ii) patient was previously healthy; (iii) EKG is normal at rest; (iv) adrenergic stimulation induces ventricular ectopy; and (v) ectopic beats become less frequent with anesthesia and opiates. Furthermore, the finding of bidirectional ventricular tachycardia is pathognomonic for CPVT [8]. The clinical diagnosis of CPVT is dependent on stress-induced symptoms, family history, catecholamine infusion, and exercise stress testing. Holter EKG monitoring can be used as a diagnostic alternative to exercise stress testing in children [4]. A genetic screening panel for pathogenic variants of RYR2, CASQ2, TRDN, TECRL, CALM1, CALM2, and CALM3 is often used to support a CPVT diagnosis [7].

CPVT-associated cardiac arrest is a circumstance in which epinephrine is contraindicated [8,9]. Administration of epinephrine for cardiac arrest patients with CPVT will provoke further ventricular tachycardia; thus, it is physiologically counterproductive and life-threatening [8,9]. Given the prevalent use of epinephrine in standardized protocols such as advanced cardiac life support (ACLS) and pediatric advanced life support (PALS), a clear understanding of CPVT and its implications for treatment should be included in ACLS and PALS certification courses.

Several case reports have reported that general anesthetics and opiates are effective treatments for cardiac arrests ascribed to CPVT. Both of these therapeutics reduce catecholaminergic stimulation, therefore halting storms of ventricular tachycardia. If IV fentanyl is used in resuscitation for this purpose, a high dosage of up to 10 µg/kg should be administered [8,9].

During the post-cardiac arrest care of CPVT patients, the administration of β-adrenergic agonists should still be avoided [10]. Vasopressors with pure α-adrenergic activity, such as phenylephrine, are safer for the treatment of hypotension in these patients [10].

Long-term management of CPVT includes lifestyle modifications, drug therapies, and surgery [11]. Survivors of CPVT-associated cardiac arrest will often receive prophylactic ICD implantation [11]. However, to minimize electrical shocks from the device, other measures should also be taken. Individuals with CPVT should be advised to avoid strenuous exercise and stressful situations. However, if stress tests reveal no signs of ventricular ectopy or arrhythmia while under treatment and a patient has been asymptomatic for at least three months, then participation in recreational sports of low-to-moderate intensity may be considered [12]. As primary arrhythmia prevention, β-adrenergic blocking agents without intrinsic sympathomimetic activity should be prescribed [11]. Nadolol is the first-choice β-blocker at a dosage of 1-2 mg/kg per day. For patients who receive recurrent ICD shocks despite β-blocker treatment, flecainide can also be prescribed. This anti-arrhythmic agent has two major mechanisms of action: blockade of cardiac Na+ channels and inhibition of cardiac ryanodine receptors. Patients who are refractory to maximal pharmacological intervention can undergo cardiac sympathetic denervation [11]. As an ultimate treatment approach for severely refractory CPVT, cardiac transplantation has been described [13].

Our case demonstrates an event of cardiac arrest in which the patient made a rare full neurological recovery. We speculate that several factors in the patient’s care led to this outcome, including bystander CPR, rapid defibrillation, avoidance of epinephrine administration, and targeted temperature management.

## Conclusions

While CPVT is an unusual cause of sudden cardiac arrest in children and adolescents, clinicians should maintain a high suspicion for this disorder in cases of unexplained pediatric cardiac arrest or syncope. Since the condition is familial, it is important to obtain from all patients with possible CPVT a detailed family history of any inexplicable sudden deaths. Clinicians should also be aware that the screening EKG is not an effective CPVT diagnostic tool, as most CPVT patients have normal baseline EKGs. In conjunction with standard resuscitation measures, when a history of CPVT is known, avoidance of epinephrine is key toward increasing the odds of successful resuscitation.
